# Promoting psycho-social wellbeing for engaging inflammatory bowel disease patients in their care: an Italian consensus statement

**DOI:** 10.1186/s40359-021-00692-6

**Published:** 2021-11-28

**Authors:** Guendalina Graffigna, Caterina Bosio, Francesco Pagnini, Eleonora Volpato, Enrica Previtali, Salvatore Leone, Ferdinando D’Amico, Alessandro Armuzzi, Silvio Danese

**Affiliations:** 1 EngageMindsHUB, Università Cattolica del Sacro Cuore Milan, Milan, Italy; 2 Amici Onlus, Milan, Italy; 3 Department of Biomedical Sciences, Humanitas University, Pieve Emanuele, Milan, Italy; 4 Fondazione Policlinico Universitario A. Gemelli IRCS, Rome, Italy; 5 IBD Center, Humanitas Research Hospital- IRCCS, Rozzano, Milan Italy

**Keywords:** Patient engagement, IBD, Psychological needs, Quality of life, Psychological support

## Abstract

**Background:**

Inflammatory bowel diseases (IBD) are remitting and relapsing diseases that mainly interest the gastrointestinal tract. IBD is associated with a condition of psycho-social discomfort that deeply compromises the quality of life and the competence of patient to be fully engaged in their self-management. As a consequence, effective care of IBD patients should include not only medical but also psychological support in order to improve patients' wellbeing. Although this, to date there is no standardized approach to promote psychological wellbeing of IBD patients in order to improve the perception of the quality of the care. To fill this gap, a consensus conference has been organized in order to define the psychosocial needs of IBD patients and to promote their engagement in daily clinical practice. This paper describes the process implemented and illustrates the recommendations deriving from it, which focus on the importance of a multidisciplinary approach in IBD management.

**Results:**

The consensus conference has been organized in three phases: (1) literature review about life experiences, engagement, and psychosocial needs of IBD patients; (2) workshops with IBD experts and patients’ representatives; (3) drafting of statements and voting. Seventy-three participants were involved in the consensus conference, and sixteen statements have been voted and approved during the consensus process.

**Conclusions:**

The main conclusion is the necessity of the early detection of – and, in case of need, intervention on- psycho-social needs of patients in order to achieve patient involvement in IBD care.

## Introduction

Inflammatory bowel diseases (IBD) are remitting and relapsing conditions that mainly interest the gastrointestinal tract. IBD include two heterogeneous diseases, ulcerative colitis (UC) and Crohn’s disease (CD) [1]. It is estimated that in Italy about 200,000 people are affected by IBD [2]. CD and UC can occur in every age of life but the highest incidence is documented between 20 and 40 years [3]. IBD are chronic diseases requiring multiple interventions and continuous monitoring and adaptation processes by patients and family members [2]. This is associated with a condition of psycho-social sufferance that prevents the affected person from living normally and deeply compromises the quality of life in terms of personal, work, and interpersonal well-being [2]. Management of IBD patients should include not only purely medical but also psychosocial aspects in order to improve patients' wellbeing and quality of life [4] and their ability to get fully engaged in self-management [5]. A growing corpus of literature has indeed demonstrated that promoting patients’ active engagement in their care process has proven to be an effective strategy to achieve a better patient's quality of life in various chronic care contexts (e.g. diabetes, kidney disease) [6] and in different phases of life (e.g. childhood or adolescence) [7]. The word “patient engagement” embodies several related concepts, including patient-centered care and shared decision-making, all of which are built on the principle of involving patients as full partners in their healthcare path [8]. Patient engagement, indeed, aims at giving a protagonist role to patients for a more efficient and effective process of care delivery. There in a lot of evidence in the literature that patient engagement is linked with a better patient self-management, a decreased use of healthcare and fewer adverse events [9]. Particularly, scientific studies have demonstrated that patients who are more engaged in their care reports better medical results [10], higher quality of life [11], higher satisfaction with their care relationship, healthier behaviors [12], more effective self-management skills [13] and treatment adherence [13]. Furthermore, some research has shown that patient engagement may contribute to a reduction of healthcare costs and to economically more sustainable organizational processes [14]. Previous research has proven that emotional adjustment and psychological wellbeing is a crucial antecedent of positive patient engagement and participation in his/ her health care process management; so is crucial to grant psychological support in order to have a more involved patient [15]. Although the undoubted role of patient engagement in the decision-making process, to date there is no standardized approach to promote the engagement and little attention is paid to social and psychological needs of subjects with IBD [16].

To fill this gap, A.M.I.C.I. association (the Italian association of IBD patients), the Higher Institute of Health and EngageMindsHUB – Consumer, Food & Health Engagement Research Center of Università Cattolica del Sacro Cuore organized a consensus conference (see Materials and methods) involving gastroenterologists, healthcare professionals expert in IBD care and IBD patients in order to define the priorities and best practices of care to ensure a self-management of the psychosocial needs of IBD patients which may improve their quality of life and engagement self-management. Here, we report the literature evidence and practical recommendations that emerged from the consensus process.

## Materials and methods

The consensus conference was conducted in accordance with the Consensus Development Program of the United States’ National Institutes of Health [17] (all methods were carried out in accordance with relevant guidelines and regulations) and was aimed to draft recommendations to answer the following three questions a priori determined by the project coordinators:

1. What are suggested strategies to ensure the management of psycho-social needs of pediatric IBD patients in order to improve their patient engagement?
2. What are suggested strategies to ensure the management of psycho-social needs of adult IBD patients in order to improve their patient engagement?
3. What e-health technologies are available for improving IBD patients’ psychological wellbeing and engagement?

The consensus conference was divided into three steps: (1) literature review on published evidence about life experiences, engagement, and psychosocial needs of IBD patients (see Appendix 2 for literature review details and Volpato et. Al. [18]); (2) workshops with IBD experts and patients representatives (held in October 2019); (3) drafting of statements and voting. The final drafting and presentation to the press was held on the 23rd and 24th of June 2020.

### Participants

A purposive selection of participants was made by following two steps: (1) identification of the categories of stakeholders/actors relevant to IBD management (see Table 2); (2) identification and enrollment—for each category—of prototypical representatives (bearers of experience in the field and/or opinion leaders in the context of reference). Due to the goal of ensuring multi-disciplinarity in the selection of experts, no fixed criteria were a priori settled to define the extent of expertise and we followed a broad inclusive concept of expertise such as the ownership of the relevant knowledge, skills and experience in the management of IBD care. Furthermore, experts have been defined as relevant key opinion leaders in their field of scientific expertise. In order to guarantee the scientific standards of the process the stakeholders involved in the project were asked to sign a conflict-of-interest statement and to accept the Consensus conference regulation. The Research Team paid close attention to guarantee the equal chance for everyone to express his/her own opinion in the workshops and meetings to discuss the collected evidence. Furthermore, according to the methodology of Consensus Conference approach, an organizational structure for the Consensus Process was set up as described in Table 1 [19]. A detailed list of participants in the different roles is provided in the Appendix 1.

**Table 1:** The roles in the consensus conference

Role	Roles
Organizing committee	It was responsible for:
	Setting the goals of the conference;
	Finding funds;
	Choosing components of the Technical-Scientific Committee (TSC);
	Drafting the protocol with the TSC;
	Disseminating news about the conference;
	Choosing components of the Panel Jury;
	Defining, in concordance with the Technical-Scientific Committee, the questions for the Panel Jury;
	Offering methodological support to the Experts for the drafting of the reports to send to the Panel Jury;
	Choosing the measuring methods of the impact of the recommendations produced
Technical-scientific committee	It involved members with scientific know how and representativeness and was responsible for:
	Cooperating with the organizing committee in the drafting of the consensus conference document;
	Defining the questions for the Panel Jury, in concordance with the Organizing Committee;
	Choosing internally its Experts and the work groups necessary to draft and present to the Jury the reports concerning the recommendations about the different questions during the conference;
	Offering methodological support to the Experts and the work groups
Panel jury	It was meant to:
	Draft a regulation of discussion in which the procedures that the Jury will apply were described,
	Evaluate and review the documents drafted from the work groups;
	Attend the meetings for discussion of the reports of the Consensus Conference;
	Discuss, review and approve the preliminary consensus document;
	Review and approve the final consensus document respecting the procedure given in the regulation
President of the jury	It had the authority to:
	Draft the regulation document and get the Jury’s members’ approval;
	Guarantee that all the Jury members receive the materials drafted by the experts and work groups by the right time;
	Coordinate the Jury and the Writing Committee during the process towards the final consensus document;
	Manage the Jury’s discussions;
	Manage the relationships with the Organization Committee and act as a medium of communication directed to the Jury;
	Attend the celebration of the Consensus Conference;
	Communicate publicly, at the end of the discussion of the Jury, the recommendations written in the final preliminary consensus document
Writing committee	Composed by members extracted from the Jury, this Committee embodies the multidisciplinary of the Panel, check and gives to the newsroom the final consensus document, following the rules agreed and subscribed in the Jury regulation
Scientific secretariat	It manages the exchange of materials and documents and information among the components and the groups of the consensus
Organizational secretariat	It manages the operative organization of the conference
Members of the expert meetings	Reviews and summarizes the evidence of the scientific literature related to the questions of the Consensus Conference. Particularly:
	Drafts a summary of the scientific evidence available;
	Drafts a summary of the information available to the public that comes from different sources, concerning the themes of the conference;
	Sends the reports made to the Jury by the agreed deadline;
	Presents the data collected during the celebration of the conference and participates to the discussion

### Literature review

A systematic search of the scientific literature was conducted in electronic databases from January 2009 to October 2019 in accordance with the approach defined by Arksey and O’Malley [20] and the PRISMA extension for scoping reviews [21]. A detailed description of the literature review process is provided in the Appendix 2 [18].

### Workshops

Five workshops were organized. One workshop addressed the technology question, while the questions about needs and interventions were each articulated in two workshops (one focused on the developmental age and one on the adult age). All workshops involved a balanced mix of IBD experts and patients’ representatives following the maximum variety coverage criterion [17]. The workshops – moderated in a non-directive approach [22]–had a scheduled duration of 4 h. The moderators ensured the equal participation of patients and caregivers advocates in all the meetings. The documents generated during the workshops were discussed, revised, and enriched by the Panel of Jury participants in order to reach a collegial approval.

### Consensus statements

Finally, the jury panel (to see the composition of the jury panel see Appendix 1) revised the workshop reports in order to elaborate the final statements of the consensus. The jury panel, discussed, drafted, voted and approved the final document. The statements included in the document were selected on the basis of a twofold criterion of (1) being approved by the majority of the panel members; (2) having received no overt expression of dissent.

## Results

Seventy-three participants were involved in the consensus conference including IBD experts from different scientific and professional fields and representatives of patient associations (Table 2).

**Table 2:** Participants to the consensus conference

Stakeholders	N (%)
Medical doctors*	27 (37)
Pediatricians	10 (14)
Psychologists	13 (18)
Nurses	5 (7)
Patients	11 (15)
Healthcare political decision makers	7 (9)
Total	73 (100)

*10 women and 17 men

The available literature evidence on this topic has also been previously reported more broadly by Volpato and colleagues [18].

### Main strategies to ensure the management of psycho-social needs of pediatric IBD patients in order to improve their patient engagement

#### Literature evidence

Psychosocial needs of pediatric patients with IBD include three main topics: transmission of information/knowledge, psychological/psychotherapeutic interventions, and promotion of psychosocial support [23].

An adequate communication of disease-related information to the patient (literacy) is associated with a better disease management with possible positive effects on coping competencies, perception of self-efficacy, and satisfaction with the services received [23, 24]. On this point, there are problems of continuity in the flow of information related to organizational dysfunctionality [25], especially in the moment of transition between medical structures dedicated to pediatric and to adult age.

Pediatric IBD patients experience a higher rate of psychological problems (e.g. depression, sense of helplessness, anxiety, family problems, and school difficulties) compared with the general population, requiring more psychological support [26–31]. Several validated psycho-diagnostic tools are available for the management of IBD, including the Pediatric Quality of Life (PedsQL) Family Impact Module [32], the KidScreen-10 [33], the IBD Specific Anxiety Scale (IBD-SAS) [34], and the Children’s Depression Inventory (CDI) [35]). Interestingly, most psychotherapeutic treatments showed to be effective in terms of clinical management of disease and psychological support to the patient [36–39].

Finally, attention should be paid to the psychosocial support to the pediatric IBD patient [40]. This could be done in various methods such as narrative exchange of experiences, self-help groups, mapping strengths and weaknesses of patients and their network of social support, development of coping and problem-solving skills, integration of peers/family contexts/care settings [41]. In this, patient associations can play a big role [42]. The transition in the management of IBD from childhood to adulthood should also be considered, supporting the integration between these phases both in terms of patient's personal identity and health organization and coordination of the operators involved [43, 44]. Importantly, the cooperation between physicians and nurses in this transition period is crucial to ensure an optimal management [45–47]. In addition, the connection between health care professionals and school/life contexts has been associated with improved patient care [48–54]. Of note, an uncomfortable social context could lead – not only to a worse physical symptoms management (tiredness, use of bathrooms…)- but also to negative behaviors such as personal isolation, feelings of embarrassment/humiliation in relationships, and difficulty in formulating requests for help [55–57]. The role of family and caregivers is relevant in the management of IBD patients during childhood, but to date it is still poorly explored [58]. On the other hand, appreciable results on caregivers’ quality of life and disease management capabilities were reported after psychological and social support interventions [59, 60]. The coordination between different professional figures (e.g. health professional, psychologist, nutritionist, and social worker) could improve the synergy in responding to specific psycho-social needs of patients (in addition to the clinical ones) and support patient engagement in the care process [61–64].

#### Final consensus statement

Based on literature evidence, attention should be given to the psychological needs of pediatric IBD patients in order to ensure their full engagement in care management and to improve their quality-of-care experience. In this scope, the experts claimed for the following recommendations.

##### IBD patients should be managed through a multidisciplinary approach

Different professionals should be involved in the management of pediatric IBD patients including physicians (gastroenterologists, radiologists, surgeons, pediatricians, rheumatologists, dermatologists, ophthalmologists, and general practitioners), nurses, psychologists, nutritionists, and social workers [65, 66]. This in order to take care not only of the medical needs of the patients but also of the psychosocial ones [67].

##### Patients with IBD should be provided with early psychological screening and psychosocial support

Patient psychology should be investigated already at the time of IBD diagnosis to promptly identify patients at risk of psychological and social distress [68]. The assessment should consider the social and psychological status of the patient in order to intervene on a targeted psychotherapeutic plan and/or on a psychosocial plan to promote adaptation and self-management, as well as to build a proactive role (engagement) in the management of disease impacting on quality of life. Patient's distress should be evaluated at each follow-up visit and/or hospital access: in case of critical psychosocial status an intervention has to be planned [69].

##### Psychosocial support should be provided to IBD patient's family/caregiver

Even if it is often reported in the clinical settings, there is still little scientific evidence about the distress of family and caregivers of IBD patients [61]. More studies are needed to deepen and to improve the interventions dedicated to them, as well as to define their role in the management of pediatric IBD patients.

##### Connection/cooperation between IBD patient’s clinical structure and school should be guaranteed

It is necessary to provide n adequate information on IBD at school and to promote collaboration between health caregivers and teaching staff to guarantee better physical symptoms management (tiredness, diarrhea…) prevent disease-related stigma and the loss of educational continuity during relapses or hospitalizations.

##### Awareness about IBD in evolutive age should be increased in the general population

Greater awareness in the general population about IBD during childhood and adolescence is necessary to limit the formation of social stigma and contain its negative effects on patients’ life [70]. A recent survey conducted by the European Federation of Crohn’s and Ulcerative Colitis Associations (EFCCA) found that patient organizations play a key role in reducing fears and worries of IBD patients [42] For this reason, close cooperation between physicians and patient associations is desirable in order to promote activities of dissemination of information about IBD and to ensure an optimal management of this chronic condition.

##### IBD patient’s transitioning from pediatrics to adulthood should be managed through the combined work of pediatric and adult experts (transitional ambulatory)

Pediatric and adult professionals should be involved in the management of patients with intermediate age to guide therapeutic decisions [71].

### Main strategies to ensure the management of psycho-social needs of adult IBD patients in order to improve their patient engagement

#### Main evidence

A higher proportion of psychological distress (e.g. anxiety and depression) is detected in adult IBD patients than in both general population and patients with other diseases [72, 73]. IBD patients may have an altered perception of their body image, resulting in a negative impact on subjective well-being [74, 75]. In addition, IBD-related fatigue is associated with a decreased quality of life [76]. Regarding human relationships, adult IBD patients tend to social withdrawal and to experience considerable difficulties in adapting to daily and working life due to the unpredictability of the disease course [77, 78].

Several psychotherapeutic and psychosocial interventions are available to achieve adequate levels of well-being and quality of life. In this context, different initiatives have been proposed including a focus on diet, physical activity, sleep [79–83], and restoration of a good social relationship [81, 82, 84–93]. Effective clinical and social management of IBD requires the active involvement of health care stakeholders and health care organizations implicated in this condition [94, 95].

To date, there is a gap between the level of information on disease provided by health professionals and the one required by patients [96–98]. An appropriate transmission of information is a prerequisite for the development of patient engagement [99]. Interestingly, engagement programs should involve not only the patient but also the family, caregivers, and the care team [94, 95].

With regard to the care team, programs have been developed to improve communication skills strengthening the physician–patient relationship [49, 96, 100]. Similarly to what found for pediatric patients, the psycho-social needs should be taken into account in a multidisciplinary perspective also in adult IBD patients in order to integrate the performances of health professionals (doctors, nurses…) and lay people (family members, caregivers, and patients' associations) [101]. Consequently, the involvement of non-medical personnel in the management of IBD patients and caregivers should be enhanced [86–89, 102]. Growing evidence shows that the physician should be accompanied by a figure (case manager) who coordinates the interactions among IBD patient, hospital, and any other parties [103]. This multidisciplinary strategy should be associated with organizational changes leading to the development of IBD unit including physicians, nurses, and psychologists to meet patients’ needs [101, 104] and ensure continuity of care [105].

#### Final consensus statement

Based on the indications offered by the literature, the experts formulated seven recommendations for the management of IBD patients.

##### An integrated approach to the adult IBD patient should be used

A multidisciplinary structured approach is needed to take care (in addition of the medical ones) of psycho-socio-assistential-existential needs of IBD patients. Dedicated and specialized nurses and psychologists should be fully integrated in IBD management unit (composed by all the members that, at various levels, address IBD).

##### Active IBD patient engagement should be promoted

Effective response to patient's psychosocial needs (in addition of the clinical ones) requires the engagement of patients, family members/caregivers, and care providers through appropriate information and support interventions.

##### IBD physician–patient communication should be enhanced

Specific attention should be paid to physician–patient communication through communication and empathy training programs to improve the effectiveness of the therapeutic relationship. Communication should be flexibly modulated in relation to critical moments and specific phases of the patient journey (diagnosis, pre and post-surgical phases, worsening of the condition…).

##### Continuity of IBD care should be monitored

It is necessary to ensure a therapeutic continuity to achieve an effective response (in addition of the clinical ones) to the psycho-social needs of patients.

##### Adult IBD patient support should be planned

For the adult patient, psychological (and, if necessary, psychotherapeutic) support should be planned and implemented, to overcome critical moments and achieve/maintain good levels of quality of life.

##### IBD patients’ family and caregiver support should be planned

More attention should be paid to family members and caregivers implementing psychological/psychosocial initiatives dedicated to them [106].

##### General knowledge about IBD should be improved

Communication interventions should be implemented and maintained in order to improve knowledge of IBD, its psycho-social impact on quality of life, and reduce the negative effects of social stigma related to IBD [107].

### What e-health technologies are available for improving IBD patients psychological wellbeing and engagement?

#### Main evidence

E-Health is a wide term, and is referred to the utilization of information and communications technologies in the healthcare world [108]. Scientific research, although in its beginnings, has already highlighted some opportunities for the application of e-health technologies in the management of chronic diseases, such IBD. Among the most important: the possibility of sharing stories and experiences to support the emotional management of one's pathology [109]; the supply of an effective support to maintain therapeutic continuity and promote the physician–patient relationship even in "remote" settings [110]. E-health technologies can also limit the spread of fake news (linked to the use of unreliable or inaccurate digital sources to search for information on a pathology) by spreading correct information [111].

Moreover, several e-health tools are available to be used in different fields and patient groups in chronic care management [111, 112]. Web-apps can provide the adult patient with useful information for self-education, monitoring of disease and side effects, as well as for therapeutic decision-making [106, 110, 113]. Telemedicine can be an effective communication channel between patient and physician outside the routine clinical practice (e.g.: unexpected events, need for unscheduled consultations in the presence of unpredictable flare-ups) [110]; more in particular, in young and adolescent patients, a telemedicine-based approach can be useful to support the achievement of skills and competencies (self-management, adherence, therapeutic relationship satisfaction…) in the transition to adulthood, saving time, costs and ensuring disease monitoring [114]. Social media can play an elective role in sharing experiences and promoting social support [114]. Finally, groups and disease-related web pages represent an important "place" for the creation of a lay network of sharing and support, in particular when physical participation is not possible or complicated [115].

#### Consensus statement

Based on the available evidence, three statements were approved with reference to new technologies.

##### Further studies on usability and effectiveness of e-health technologies in IBD management should be promoted

The use of e-health technologies, even if the first pieces of evidence are encouraging, requires further research with respect to usability and effectiveness.

##### Personalized e-health interventions in IBD management should be planned according to the characteristics of the target audience

The e-health technologies should be chosen on the basis of available tools, patient targets, and combination between target and tool specificity (e.g.: a too sophisticated technology for an old or a low-cultured patient could not work being too difficult for him to use).

##### E-health interventions in IBD management should be planned according to the demonstrated utility

The choice of specific e-health technologies should be justified by its proven usefulness (e.g. sharing of experiences among patients, therapeutic continuity, and prevention of fake news).

### The statements at a glance

The whole of the statements of this consensus conference is also summarized in the Table 3.

**Table 3:** The statements of the consensus conference

Main strategies to ensure the management of psycho-social needs of pediatric IBD patients in order to improve their patient engagement	IBD patients should be managed through a multidisciplinary approach
	Patients with IBD should be provided with early psychological screening and psychosocial support
	Psychosocial support should be provided to the IBD patient's family/caregiver
	Connection/cooperation between IBD patient’s clinical structure and the school should be guaranteed
	Awareness of IBD in the general population should be increased
	IBD patient’s transitioning from pediatrics to adulthood should be managed through the combined work of pediatric and adult experts (transitional ambulatory)
Main strategies to ensure the management of psycho-social needs of adult IBD patients in order to improve their patient engagement	An integrated approach to the adult IBD patient should be used
	Active IBD patient engagement should be promoted
	IBD Physician–patient communication should be enhanced
	Continuity of IBD care should be monitored
	Adult IBD patient support should be planned
	IBD patients’ family and caregiver support should be planned
	General knowledge about IBD should be improved
What e-health technologies are available for improving IBD patients psychological wellbeing and engagement?	Further studies on usability and effectiveness of e-health technologies in IBD management should be promoted
	Personalized e-health interventions in IBD management should be planned according to the characteristics of the target audience
	E-health interventions in IBD management should be planned according to the demonstrated utility

## Discussion

IBD disease has an undoubted impact on patients’ quality of life with important consequences at psychosocial level [116]. However, psychosocial support for IBD care is still underestimated and there is no clear consensus on priorities for interventions. In addition, although patient engagement is a crucial target in chronic care, it often appears to be an abstract principle and it is not applied to clinical practice^117^. Based on these considerations, we conducted a consensus conference to animate a scientific, multidisciplinary, and inclusive debate in which IBD patients could participate together with scientists and healthcare professional with the final aim of improving psycho-social wellbeing and engagement of IBD patients. This project was the occasion to bridge expertise, visions, and concerns from multiples scientific disciplines and with the subjective and crucial expertise of the patients about their needs and priorities. So, a co-constructive and collaborative endeavor was enabled along the whole consensus process and this in itself constitutes a best practice for innovation of IBD care practices. The consensus statements emerged offer a complex and integrated framework aimed to track the specificities of the psycho-social unmet needs of patients along their life-cycle and in the different phases of the patient journey. Early detection of psycho-social needs of patients is the first priority for achieving a truly patient centered and participate approach to IBD care. A second crucial step concerns the design and implementation of an organic approach to the promotion of patient and caregiver/family engagement. This approach requires not only the development of an effective information activity (literacy), but also of adequate psychological and psychosocial initiatives aimed at fostering active involvement in the management of IBD. To this end (third step), it is also necessary to educate and sensitize all the different healthcare professionals involved in the IBD care in order to support not only the Engagement of the patient/caregiver but also of the healthcare organizations in charge of the management of the disease (with specific attention to the hospital-territory connection). Fourth, a better sensitive and personalized use of the different digital health technologies today available can contribute to achieve these goals, but in the light of further education activities about a more proficient and aware use of such devices. Finally, an intervention on public opinion, stakeholders and health policy makers is important to improve the representation and management of IBD in society, free from the prejudices of the IBD stigma. In this perspective, the role of patient associations may play a fundamental role in such transformation process as a catalyst of the cultural shift required both from the healthcare professionals’ and the IBD patients’ side.

## Limitations

Even if the consensus Statement process has been broad, deep and inclusive there were a few limitations. First, any generalizations from the literature review or workshops must be done carefully being that they represent the specific experience and perspectives of the individuals included in the process, and a forthcoming replica of the consensus process in a wider international scenario would be useful. Another limitation is connected to the national nature of this Consensus Conference: the habits of clinical practice of IBD may indeed change in different countries and local communities. Nonetheless, the participating experts have a reputable international clinical and scientific know how on the theme of the conference. Finally, these recommendations are not meant to be paradigmatic standards of practice. They are a scientific outline of best practices in the IBD sector. These recommendations should be taken as inspiring concepts to promote an effective engagement eco-system in IBD. Indeed, the most important stage of the process is the application of this principles in the day by day IBD practice through use of validated scientific instruments and practices.

## Conclusions

Going beyond the indications illustrated and discussed in the previous paragraphs, a final remark can be made about the "usability" of the consensus conference as a tool to facilitate the promotion the psychological wellbeing and the engagement of patients in the context of IBD management.

Beyond the appropriate improvements of the process (see “Limitations” paragraph), we believe that three advantages of the consensus conference method can be electively recognized at the operational level: (a) the possibility of designing interventions closely related to the indications offered by scientific research; (b) the possibility of articulating at the organizational level the actors and the practices involved in the process of promoting engagement, by integrating the medical-clinical perspective with the psycho-social one; (c) finally, the possibility of building a shared course of action that is inclusive of all the actors (experts and lay people) involved in the process. We plan to disseminate these results through conferences, congresses and publications.

## Data Availability

All the data and materials are provided or referenced in the paper.

## Appendix 1: Consensus conference organizational chart

*Institutional representatives*: A.M.I.C.I. Onlus; EngageMinds HUB—Consumer, Food & Health Engagement Research Center Sacred Heart Catholic University—Milan; Istituto Superiore di Sanità.

*Promoting committee:* S. Leone, A.M.I.C.I. Onlus; E. Previtali A.M.I.C.I. Onlus; G. Graffigna, EngageMinds HUB, Consumer, Food & Health Engagement Research Center, Sacred Heart Catholic University; M. Boirivant, Istituto Superiore di Sanità; S. Brusaferro, Istituto Superiore di Sanità.

### Technical and scientific committee

- Tonino Aceti|FNOPI (National Federation of Orders of Nursing Professions)
- Alessandro Armuzzi|IG-IBD (Italian Group for the Study of Inflammatory Bowel Disease)
- Livia Biancone| “Tor Vergata" University of Rome
- Piero Cai|Policlinico San Martino IRCCS Hospital
- Elena Carnevali|Social Affairs Commission Chamber of Deputies
- Liliana Coppola|Region of Lombardy
- Silvio Danese|Humanitas Research Hospital, Rozzano|Past president ECCO
- Luigi Dall’Oglio|Pediatric Hospital Bambino Gesù, Roma
- Santo Di Nuovo|AIP (Italian Association of Psychology)
- Giulio Gallera|Region of Lombardy
- Maria Alessandra Gallone|Republic Senate
- Antonio Gaudioso|Secretary General Cittadinanzattiva
- Antonio Gasbarrini|Fondazione Policlinico Universitario Agostino Gemelli IRCCS Università Cattolica del Sacro Cuore, Rome
- Fulvio Giardina|CNOP (National Council Order Psychologists)
- David Lazzari|CNOP President
- Paolo Lionetti|Full Professor of Pediatrics University of Florence, Head of Gastroenterology and Nutrition SOD Meyer Pediatric Hospital
- Beatrice Mazzoleni|IPASVI (Professional Nurses, Health Care Assistants, Child Care Workers)
- Enrico Molinari|Sacred Heart Catholic University, Auxological Institute of Milan
- Giovanni Muttillo|IPASVI (Professional Nurses, Health Care Assistants, Child Care Workers)
- Gilberto Poggioli|Alma Mater Studiorum University of Bologna, Policlinico Sant'Orsola Bologna
- Claudio Romano|University of Messina
- Maria Rizzotti|Republic Senate
- Gabriele Roveron|AIOSS (Italian Association of Health Operators of Stomatherapy)

### Work groups

#### Group on psychosocial support needs in the child with IBD

- Marina Aloi|La Sapienza University, Rome
- Claudia Canaletti|A.M.I.C.I. Onlus
- Eleonora Geccherle|Sacro Cuore Don Calabria" Hospital Negrar” (VR)
- Giammarco Mocci|G. Brotzu" Hospital, Cagliari
- Lorenzo Norsa|ASST Hospital Pope John XXIII, Bergamo
- Francesca Maria Onidi|G. Brotzu" Hospital, Cagliari
- Floriana Perillo|A.M.I.C.I. Onlus
- Sonia Storoni|A.M.I.C.I. Onlus

### Group on psychosocial interventions to support the child with IBD

- Enrica Atzori|A.M.I.C.I. Onlus
- Matteo Bramuzzo|IRCCS «Burlo Garofolo», Trieste
- Gionata Fiorino|Humanitas Research Hospital, Rozzano
- Simona Gatti|Azienda Ospedaliero Universitaria Ospedali Riuniti of Ancona
- Antonino Morabito|Meyer University Hospital, Florence
- Maria Simonetta Spada|ASST Hospital Pope John XXIII, Bergamo
- Claudia Venditti|A.M.I.C.I. Onlus

### Group on psychosocial support needs in adults with IBD

- Giovanna Artioli|IPASVI (Professional Nurses, Health Care Assistants, Child Care Workers)
- Concetta Balzotti|A.M.I.C.I. Onlus
- Luciano Bertolusso|FIMMG (Italian Federation of General Practitioners)
- Gaia Campanale|ASST Santi Paolo e Carlo, University of Milan
- Federico Colombo|IMIPSI (Milanese Institute of Cognitive Behavioral Psychotherapy)
- Renata D’Incà|Hospital of Padua
- Manuela Pes|A.M.I.C.I. Onlus
- Simona Radice|Humanitas Research Hospital, Rozzano
- Elena Vegni|State University of Milan

### Group on psychosocial interventions to support the adult with IBD

- Alessandro Agostini|DIMES (Department of Specialistic, Diagnostic, Experimental Medicine), University of Bologna
- Flavio Caprioli|Fondazione IRCCS Ca’ Granda Ospedale Policlinico di Milano
- Giuseppe Coppolino|A.M.I.C.I. Onlus
- Antonio Di Sabatino|Fondazione IRCCS Policlinico San Matteo Pavia
- Maria Galanti|A.M.I.C.I. Onlus
- Daniele Napolitano|Agostino Gemelli Foundation University Hospital, Rome
- Gaspare Solina|Azienda Ospedaliera Ospedali Riuniti Villa Sofia Cervello, Palermo
- Marinella Sommaruga|CNOP (Consiglio Nazionale Ordine Psicologi)

### Group on the definition of technology in the psychosocial care of the patient with IBD

- Andrea Dessì|A.M.I.C.I. Onlus
- Anna Kohn|IG-IBD (Italian Group for the Study of Inflammatory Bowel Disease)
- Filippo Mocciaro|ARNAS Civico Di Cristina, Palermo
- Claudia Repetto|Sacred Heart Catholic University
- Luigi Sofo|Fondazione Policlinico Universitario Agostino Gemelli IRCCS Università cattolica del Sacro Cuore, Rome
- Antonino Spinelli|Humanitas Research Hospital, Rozzano

### Jury panel

- Patrizia Alvisi|SIGENP (Italian Society of Pediatric Gastroenterology, Hepatology and Nutrition)
- Gianluca Castelnuovo|AIP (Italian Association of Psychology)
- Antonella Celano|APMARR (National Association of People with Rheumatologic and Rare Diseases)
- Marco Daperno|General Secretary IG-IBD|Vice-Chairman of the Jury Panel
- Ferdinando Ficari|Careggi University Hospital, Florence
- Anna Lisa Mandorino|Deputy Secretary Cittadinanzattiva|Secretary of the Jury Panel
- Paolo Michielin|University of Padua, AIAMC (Italian Association of Behavior Analysis and Modification and Behavioral and Cognitive Therapy)
- Giuseppe Parisi|SIPeM (Italian Society of Medical Pedagogy)
- Paola Pisanti|Ministry of Health Chronic Disease Consultant|Chairman of the Jury Panel
- Alessandra Tongiorgi|Pisana University Hospital
- Silvia Tonolo|ANMAR (National Association of Rheumatic Diseases)

### Writing committee: A. Celano, A. Tongiorgi

- Antonella Celano|APMARR (National Association of People with Rheumatologic and Rare Diseases)
- Alessandra Tongiorgi|Pisana University Hospital

### Methodological support

- **F. Pagnini|**Sacred Heart Catholic University – Milan (Coordinator)
- C. Bosio|EngageMinds HUB—Sacred Heart Catholic University
- E. Volpato|Sacred Heart Catholic University—Milan

### Scientific-organizational coordination center

#### Scientific secretariat

- **C. Bosio| **EngageMinds HUB—Sacred Heart Catholic University

#### Organizational secretariat

- M. Rizzi|A.M.I.C.I. Onlus
- N. Fiumanò|A.M.I.C.I. Onlus
- C. Ranghetti|A.M.I.C.I. Onlus

## Appendix 2: The literature review process

**Table:**

Method	Literature review according to the principles of scoping review methodology [20, 21]
Search string	("IBD*" OR "inflammatory bowel disease*" OR "Crohn*" OR "ulcerative colitis") AND ("Psych*" OR "social*" OR "famil*" OR "Engagement" OR "empowerment") AND ( "intervention" OR "trial" OR "program" OR "strategy*" OR "counsel*" OR "treatment" OR "support “)
Queried databases	Health and Life Sciences, Social Sciences and Medical Sciences, such as PubMed, Medline, Embase, Scopus, Cochrane, Web of Science, PsychInfo
Inclusion criteria	Years considered: 2009–2019; language: English (recognized language of international scientific debate); types of articles: qualitative or quantitative studies published in peer-reviewed journals, commentaries, letters, editorials; ethical aspects: studies approved by Ethics Committees; age groups considered: pediatric (< 18 years), adult (≥ 18 years and < 60 years), and elderly patients (≥ 60 years); population: chronic inflammatory bowel disease (IBD), ulcerative colitis (UC), Crohn's disease (CD); focus of the literature: studies that explicitly discuss priority social care and psychological needs, best practices for a social care intake, the concept of Patient Engagement, studies on social and/or psychological strategies that value the patient's point of view and experience, studies that present a clear theoretical framework on patient experience, studies that focus on the experience of using care, quality of life indicators and monitoring, in the context of the chronic diseases considered
Analysis	Structured analysis grid following a mixed-methods approach given the qualitative and quantitative nature of the studies included: (1) methodological characteristics of the study (e.g., year of publication, country of first author, study design, no. of participants); (2) characteristics of the participants (e.g., diagnosis, age range, presence of caregivers); (3) characteristics of the social and psychological needs (e.g., for which transition phase, type of need expressed, theoretical framework considered); (4) characteristics of the social and/or psychological strategy (if present: type of strategy, tools used, group or individual, theoretical foundations thereof). (4) characteristics of the social and/or psychological strategy (if any: type of strategy, tools used, group or individual, theoretical foundations of the same); (5) results obtained (e.g. outcomes measured, method of evaluating results, overall results achieved)

## Abbreviations

A.M.I.C.I. Onlus: Associazione Nazionale per le Malattie Infiammatorie Croniche dell'Intestino
IBD: Inflammatory bowel disease
IG-IBD: Italian group for chronic inflammatory bowel diseases
ISS: Istituto Superiore di Sanità
